# A cross-country review of strategies of the German development cooperation to strengthen human resources

**DOI:** 10.1186/1478-4491-7-46

**Published:** 2009-06-05

**Authors:** Ricarda Windisch, Kaspar Wyss, Helen Prytherch

**Affiliations:** 1 Swiss Centre for International Health, Swiss Tropical Institute, Basel, Switzerland

## Abstract

**Background:**

Recent years have seen growing awareness of the importance of human resources for health in health systems and with it an intensifying of the international and national policies in place to steer a response. This paper looks at how governments and donors in five countries – Cameroon, Indonesia, Malawi, Rwanda and Tanzania – have translated such policies into action. More detailed information with regard to initiatives of German development cooperation brings additional depth to the range and entry doors of human resources for health initiatives from the perspective of donor cooperation.

**Methods:**

This qualitative study systematically presents different approaches and stages to human resources for health development in a cross-country comparison. An important reference to capture implementation at country level was grey literature such as policy documents and programme reports. In-depth interviews along a predefined grid with national and international stakeholders in the five countries provided information on issues related to human resources for health policy processes and implementation.

**Results:**

All five countries have institutional entities in place and have drawn up national policies to address human resources for health. Only some of the countries have translated policies into strategies with defined targets and national programmes with budgets and operational plans. Traditional approaches of supporting training for individual health professionals continue to dominate. In some cases partners have played an advocacy and technical role to promote human resources for health development at the highest political levels, but usually they still focus on the provision of ad hoc training within their programmes, which may not be in line with national human resources for health development efforts or may even be counterproductive to them. Countries that face an emergency, such as Malawi, have intensified their efforts within a relatively short time and by using donor funding support also through new initiatives such as the Global Fund to Fight AIDS, Tuberculosis and Malaria.

**Conclusion:**

The country case studies illustrate the range of initiatives that have surged in recent years and some main trends in terms of donor initiatives. Though attention and priority attributed to human resources for health is increasing, there is still a focus on single initiatives and programmes. This can be explained in part by the complexity of the issue, and in part by its need to be addressed through a long-term approach including public sector and salary reforms that go beyond the health sector.

## Background

Today there are increased awareness and consensus that strengthening human resources for health (HRH) entails a broad set of reforms that go beyond the training of health staff. Important documents include the World Health Organization's (WHO) *World health report 2006 *and the Joint Learning Initiative's *Human resources for health: overcoming the crisis*, 2004 [1,2]. Moreover, some global health initiatives such as the Global Fund to Fight AIDS, Tuberculosis and Malaria have started to adapt their agenda to account for the need to strengthen HRH [3].

This increase of awareness has, however, only to a limited extent turned into broader support by bilateral and multilateral agencies to strengthen HRH at country level. There is still little information on how countries actually address HRH development [4]. There is also little information on initiatives and roles of donor cooperation in the context of HRH development.

To address these issues, this paper reviews country initiatives for HRH in five low-income countries: Cameroon, Indonesia, Malawi, Rwanda and Tanzania. It outlines the situation with regard to HRH in general in those countries and then provides additional detail on initiatives by German development cooperation. German development cooperation was chosen as a relatively large donor that has initiated a stronger focus on initiatives to strengthen HRH.

With regard to the institutions of German development cooperation subject to this review: German Technical Cooperation (GTZ), with its 67 country offices, is the main technical implementing agency on behalf of the German Federal Ministry for Economic Cooperation and Development (BMZ). In the programme-based approach, GTZ focuses upon technical assistance, while the German Development Bank (*Kreditanstalt für Wiederaufbau*) complements with financial assistance through a mix of modalities including budget support or basket fund contributions. The German Development Service (DED) primarily places expatriate technical expertise at regional and district level in around 40 partner countries. The main objective of the institution Capacity Building International (InWEnt) is to support capacity building in developing countries, including building up and supporting training institutions, facilitating continuous and on-the-job training, etc. The Centrum for International Migration (CIM) is a joint operation of the GTZ and the German Federal Employment Agency (BA) that enables developing countries to recruit senior and qualified staff from the European Union at a local salary that is topped up by CIM. Not a typical actor in the frame of development cooperation, the German Academic Exchange Service (DAAD) plays a role with regard to HRH in developing countries, given that it facilitates study and research stays in Germany and supports reintegration of scholars in low-income countries through incentives such as continuous education and alumni networks.

Countries included in this study vary considerably with regard to HRH density. Malawi has by far the lowest rates of physician density, followed by Tanzania and then Rwanda (Table 1). Cameroon and Indonesia are facing less critical shortages. These two countries, followed by Tanzania, also have the highest aggregated rates of nurses and other auxiliary health workers (Table 1).

**Table 1:** Health workers in public services per 1000 people

**Indicator (per 1000 population)**	**Cameroon** **(2004)**	**Indonesia** **(2003)**	**Malawi** **(2004)**	**Rwanda** **(2004)**	**Tanzania** **(2002)**
Physicians	0.19	0.13	0.02	0.05	0.02

Nurses	1.60	0.57	0.59	0.42	0.30

Midwives	0.00	0.25	N.A.	0.01	0.07

Other health workers	0.00	0.10	0.06	0.06	0.82

Health management and support workers	0.36	1.04	N.A.	0.10	0.02

Source: WHO Statistical Information System (WHOSIS), consulted online at 12.02.08

All five countries face similar causes for their shortages, including brain drain to the private sector and other countries, low salaries, poor working conditions and insufficient training capacities. Cameroon, Malawi and Tanzania were particularly affected by public sector freezes as part of structural adjustment programmes initiated and supported by the World Bank and the International Monetary Fund during the 1980s and 1990s. Large-scale emigration of skilled staff and loss of health staff compounded by a high TB and AIDS burden is most pronounced in Malawi [5-7]. All five countries face unequal staff densities between rural and urban areas. In Rwanda, an estimated 75% of doctors and 50% of nurses currently work in Kigali. In Tanzania, where 66% to 80% of the total population live in rural areas, only one third of physicians work in rural areas [8].

## Methods

We selected the five countries as they represent different regions (Asia and Central and East Africa) with diverse settings and challenges for addressing HRH. In addition, they are focus countries of German development health programmes. The information was assembled through a literature review and by interviewing more than 40 representatives from the five agencies working in the five study countries in the frame of German development cooperation; representatives from other bilateral and multilateral agencies were also interviewed. All interviews were based on a guide to assure standardized data across countries. Interviews were conducted between September and November 2006 by the authors of this study. The information was complemented by a literature review including both peer-reviewed and grey literature. Grey literature included national policy documents, programme reports and evaluations to assess priority setting and implementation of initiatives at country level. The corpus of grey literature was assembled primarily through the network of country representatives interviewed in the course of this study.

The largely qualitative data was transcribed and analysed along a pre-established grid. Interventions were grouped according to stages and components that together constitute a national response to HRH development. This included looking at how far country initiatives include setting up institutions and policies at national and international level, pre-service and continuous training and other financial and non-financial incentives, as well as increasing the quantity of staff through recruitment of external staff and reintegration of national health personnel from abroad. Results and outcomes of these initiatives are structured along this framework and are presented in the next section.

## Results and discussion

### Policy context

All countries except Malawi have decentralized the management of HRH. In Indonesia decentralization contributed to an increase in regional inequity of available health staff, with a range of one- to five-fold due to unequal district planning capacities and incentive structures [9,10]. Initiatives in Malawi were to strengthen regional technical support structures for HRH. The German Cooperation has contributed to this initiative by placing regional support staff.

Most of the five countries studied have extensive private sectors. In Malawi, 37% of health service provision isthrough church-based health facilities under the Christian HealthAssociation of Malawi (CHAM). Working conditions at CHAM are generally judged as better than in the public sector [11]. Similarly, Cameroon has an important private health sector, partly originating from an economic crisis that triggered a public sector employment freeze and a 50% reduction of public salaries [12-14]. Skilled health workers trained in the public sector often remain unemployed or seek jobs in the private sector [15]. The national effort to increase salaries for health workers in Malawi included provision for CHAM staff.

Overall, this study has found few important initiatives in the five countries to address the issue of public/private sector regulation. One exception is the SETP programme, explained below, which aims to improve nurse training through public-private partnership between the Malawian government and CHAM, which owns many of the training institutions [5]. Another example is in Tanzania, where GTZ supported the Ministry of Health's effort to address dual practice in private and public services by formally allowing higher cadres to work in both sectors [16].

All five countries identified HRH development as a significant component of health sector reforms and established HRH taskforces or boards within the ministry of health (MoH). How far HRH policies are based on needs assessments and are budgeted and translated into operational targets differs between the countries. In Tanzania, where HRH is one of the nine strategies of the health sector reform programme, a strategic plan to address HRH was under development at the time of the review. Cameroon has only recently started to attribute some priority to HRH within its health policy; HRH is intended to receive more attention within the health policy for 2008–2015. The country has developed strategies for training, career development and a standardized distribution of staff, but those are largely not known and implemented at district level.

Malawi, Indonesia and Tanzania have undertaken extensive studies to assess HRH needs. This has usually been supported by international partners: in Indonesia by German development cooperation; in Tanzania by McKinsey & Partners, among others [17]. In both countries, German development cooperation and other partners have played an advocacy role to promote the development of HRH. In Indonesia and Malawi, needs assessments were translated into strategic plans with defined targets to increase quantity, quality and distribution of staff. In Malawi the MoH has initiated two major national programmes to address HRH, a six-year Emergency Pre-Service Training Plan (SETP) in 2001 and the Emergency Human Resource Programme (EHRP) in 2004, with funding mainly from bilateral development partners and the Global Fund to Fight AIDS, Tuberculosis and Malaria [18]. In Indonesia, external support to HRH is led by German development cooperation's implementing a health sector support programme with a specific focus on HRH. A comprehensive situation analysis defines the different entry doors, such as policy and planning issues, as well as pre- and in-service education. Moreover, German development cooperation in Indonesia has the government mandate to facilitate coordination and streamline processes across different departments (health, education, and finance) at national and regional level.

All five countries studied have started to develop a human resources information system (HRIS). The HRIS in Malawi is currently developed with financial support from the World Bank [19]. In Tanzania, support is through the Capacity Project funded by the United States Agency for International Development (USAID) [4]. In Cameroon it is the German Technical Cooperation that supports the development of software applications for HRH management to be used by the HRH department of the MoH. In Indonesia a HRIS is being developed with joint support of WHO and GTZ.

The need to increase financial support for HRH is slowly gaining ground. In Malawi funding for HRH is channelled via the Sector Wide Approach (SWAp); the sector further benefited from a USD 40 million reallocation of Global Fund monies from HIV to HRH in 2005. This sector- and system-wide approach is not supported by all funding agencies; at the time of the review, GAVI, for example, was supporting primarily training relevant to its own vertical programme. Other countries beyond Malawi have also started to consider their SWAps as a vehicle to facilitate a comprehensive system-wide response to HRH.

Malawi, given its elevated need to address HRH, has triggered intensified efforts among international partners in this area. In the other countries where shortages appear to be less severe, including Cameroon, Tanzania and Rwanda, the donor response to HRH development is less specific, consisting mainly of training approaches as part of different programmes to strengthen the health sector. In general there is relatively little focus on more comprehensive responses to HRH development.

International partners in the five countries have started to advocate wide-sweeping approaches to address underlying capacity weaknesses in health systems. In Rwanda, for example, as part of the health SWAp, a "basket fund for human resources in health" has been newly developed. In several of the countries, German development cooperation provides technical assistance on HRH to the ministry of health through SWAp arrangements and participates in the health sector's human resources technical working group.

### Salary levels and other incentives

Low salaries in the public sector and a lack of career development prospects, other incentives and good working conditions are challenges the countries face to both retain health staff and to correct urban-rural imbalances. For reasons of sustainability and risk of fragmentation, international partners have tended largely not to address those issues. The following section presents country initiatives to implement incentive schemes, including the relatively few areas of donor support.

In Malawi, a 52% salary top-up for publichealth workers has been financed through donor funding via the SWAp [20]. Difficulties of that salary reform included discontent triggered by different conditions and unclear expectations regarding the scale of top-ups. When public salaries were raised in Tanzania in 2006, discontent was triggered mainly by not considering the significant sector of church-owned facilities. Discontent among those excluded was also an issue of a selected accelerated salary enhancement scheme that focused mainly on managers [21].

Compared to the other countries studied, Malawi has initiated relatively extensive sets of incentive schemes in recent years to retain health workers both in the public and private health sector. Government incentives include freebasic and postgraduate training, greater job security compared to the private sector and a number of smaller incentives, such as free meals in some government facilities for health workers while on duty.

Incentives for higher health cadres in the private sector include schoolfees for their children, salary top-ups and other allowances such as transport, hardship or duty allowance. Together, those incentives can double the take-home pay [11]. Lower cadres in some private facilities may receive transport togo shopping, free uniforms, housing and easy access to loans [22].

One district (Thyolo), with the support of localgovernment and Médecins Sans Frontières (MSF), provides a monthly performance-linked monetary incentive as well as access to antiretroviral treatment for health staff and their families [23]. Another district (Blantyre) uses a rotation system of midwives between rural and urban areas [22].

Perceptions regarding any impact of these incentives differ. Some argue that incentives do not address important issues such as career development of nurses. A general view is that salary increases, especially with regard to the lower cadres, are too low to make a difference to reduce emigration. Salary top-ups and other incentives may have attractedsome paramedics who had retired or resigned, but were not sufficient to retain doctors and registered nurses [24].

Tanzania has set a range of initiatives that aim at increasing recognition of primary health care workers and retaining them. Incentives include introducing or improving supportive supervision, performance appraisal, responsive options for careerdevelopment and more transparent promotion processes [25]. Also, Indonesia has in recent years initiated a set of incentive structures including performance improvement models for nurses and midwives, as well as financial incentives for specialists to work in public hospitals instead of private practice [12].

Looking at the role of international partners other than in Malawi, support for national salary reforms still appears to be regarded as a government domain where donor contributions may be problematic if not sustained. The review gained anecdotal feedback that expressed ongoing concern about the distorting role of salaries paid by international organizations and the payment of per diems and other indirect incentives outside the public system.

### Pre-service and in-service training

The following illustrates a spectrum of country initiatives to address pre-service and continuous training, showing the different stages per country.

Indonesia in particular is undertaking significant reform of its pre-service health education [12,26]. Training centres were developed for paramedical disciplines at provincial level to promote additional deployment of village health workers at community level. WHO and the World Bank supported reforming health education to better address public health problems. This included coordination of three different stakeholders involved in pre-service education, including the MoH, the Ministry of National Education (MONE), and the Indonesian Medical Association (IMA). Also, the German HRH programme supports reforms of pre-service and continuous training at district level that are mandated by the Indonesian government as pilots for potential scale-up. For example, one initiative was to strengthen pedagogical approaches, including training of trainers concepts within 18 nursing training schools. In the area of continuous training, Capacity Building International (InWEnt) in cooperation with a national public health school, has been implementing a district health management course since 2004. The approach implied training a pool of 20 local trainers. Two years after 2004, approximately 350 participants had graduated from the course.

The main objective of the SETP in Malawi, one of two larger HRH programmes initiated in 2001 with funding from bilateral and multilateral partners including the Global Fund, is to improve nurse training institutions. The project is a cooperation between the government and CHAM [5]. While donor agencies, including the Interchurch Organisation forDevelopment Cooperation (ICCO), GTZ and Norwegian Church Aid (NCA) financed salary top-ups and a bonding arrangement where tutors worked for two years in the training institutions in return for the payment of further study fees, the government met theoperating costs and improved infrastructure oftraining facilities andstudent accommodation. The initiative resulted in an increased number of tutors and survival of nurse training institutions that before had faced closure. Moreover, new degree courses could be set up, given that the programme invested in new laboratories at the College ofMedicine [5]. Some sources appear to show that SETP has reduced emigration of nurses [24]. Between 2003 and 2006, the number of graduates increased fourfold. In 2006 the target was to train 3000 nurses per year; some 1500 nurses were actually trained [12,27].

Looking at the cases studied, countries hardly have a defined and regulated policy on in-service education. A perception is that in the absence of such regulation, access may be determined by interest groups. In Malawi to address this issue, the College of Medicine with the support of the CIM has submitted a concept for continuous advanced training. Tanzania has a policy for continuous training and career development for the public sector, including health workers; however it largely excludes the lower clinical cadres. A perception was that continuous training in general suffers from a lack of integration and recognition within the public sector. Moreover, since training options depend on programmes and sectors financed by different partners, they often lack coordination and balance.

In Cameroon, donor support still focuses more on a traditional approach of single training initiatives as part of respective programme areas. German development cooperation in Cameroon implements a range of such training. KfW, for example, provides technical training to doctors and nurses as part of its investment to technical medical equipment. Training initiatives of GTZ and InWEnt address different areas within the health sector, such as HIV, TB and quality management. Approaches in Rwanda have taken a partly broader approach, including promotion of training at national teaching institutions and health management training at hospital and district level.

Looking at the overall picture of donor support in the area of pre-service and continuous training, international partners have started financially and technically to support pre-service and continuous national training institutions. Nevertheless, they usually still focus on a range of individual training sessions provided as capacity building for their programmes. This is despite an increased awareness that they do not necessarily imply a sustainable approach to capacity building and often address only a small area within HRH development. In Malawi, for example, despite the country's relatively advanced level of donor support to HRH, one of the most frequent contributions by many development partners such as the African Development Bank, WHO and USAID still consists of facilitating and financing on-the-job training.

### Recruitment of external staff

International expertise to meet gaps in developing countries is usually financed through donor agencies with the objective of filling single expert posts rather than aiming at country coverage. Drawbacks can include the lack of sustainability and limited ownership at country level. The latter concern is addressed within the CIM approach: while the government defines the required post and pays a local salary, German development cooperation via CIM provides a top-up to attract international expertise that isn't available within the country.

Of the countries reviewed, Malawi stands out as having followed a policy of gap filling for physicians to meet shortages in theshort term. Placing external staff is regarded as one response to an emergency "requiring exceptional measures that might otherwise be dismissed as unsustainable"[28]. External support is mainly through Voluntary Service Overseas (VSO), CIM volunteers and United Nations Volunteers and financed through the SWAp.

The medical personnel from abroad usually have additional responsibilities to transfer capacities. Staff employed under CIM are encouraged to invest about 50% of work time in teaching. Some friction was caused by differing medical cultures and remuneration levels at the beginning. Coordination between different sending agencies was another issue that has started to be addressed. To address sustainability concerns of the gap filling approach, the MoH with support from German development cooperation has started to develop a strategy for longer term gap-filling of national and international medical staff.

### Migration and reintegration

An area where international partners and industrialized countries may have an important role to play is in mitigating brain drain and supporting return and reintegration of health staff from developing countries who have worked or trained in industrialized countries. CIM supports specialists who been working in Germany to return to public service in their home countries through its "Return and Reintegrate Programme". Support includes exploring the transferability of qualifications between Germany and the country concerned and providing transport subsidies and salary top-ups for up to two years to ease reintegration.

There are approximated 600 to 700 returns via CIM per year, of whom 50 to 60 are health professionals. Indonesia alone has approximately 20 health specialists returning every year via CIM. Recognition of German medical training by Indonesian accreditation bodies was a main barrier initially and is currently being addressed. A similar programme targeting Cameroonians is implemented jointly by CIM and WUS (Work for University Services in Wiesbaden, Germany).

However, support for such return programmes in the context of further education can present a challenge. The experience of German development cooperation in Malawi was that physicians it supported to participate in postgraduate public health training seldom return to their posts in reality, despite the existence of bonding arrangements.

In Malawi, advocacy was undertaken to see a reform of nurse training into a less exportable and more country-specific qualification based upon an analysis of workload. However, the attempt was blocked by the Nursing Council and represents the complexity of reaching consensus for international migration in the context of differing perspectives, needs and rights. Moreover, though the United Kingdom's National Health Service no longer seeks to recruit health staff from Malawi, the private health sector continues to try.

## Conclusion

This study's findings show a clustering of countries according to how far they implement different components of an HRH development strategy. In Malawi, where the need to address HRH has become urgent, the introduction of a comprehensive approach comprising a broad range of initiatives is already under way. Tanzania would seem to be following this pattern.

Countries with less immediately apparent HRH needs, including Rwanda and Cameroon, have only recently started to attribute more priority to HRH. Initial activities tend to include stating HRH to be a policy priority and establishing a task group. More advanced stages include the translation of policies into strategies that may be more or less elaborated with regard to operational details.

All five countries have an HRH policy and have started to develop an HRIS. Only Malawi and Indonesia have a funded strategy with defined targets. Training continues to be the most frequently cited HRD approach. Only those countries with more advanced HRH efforts have started to implement sets of incentives to retain staff. Strategies for coordinating continuous training and linking them to career development and salary increments remain relatively neglected. The same is true of issues related to recruitment and planning capacities. A frequent drawback to addressing those issues is central level and district planning capacity to deal with the complex parallel public sector reforms often needed to ensure effective and sustained implementation of issues related to HRH.

The examples given above illustrate the range of initiatives that has surged in recent years and some main trends in terms of donor initiatives. One observation is that, though attention and priority attributed to HRH is increasing, there is still a focus on single initiatives and programmes. Partly this can be explained by the complexity of the issue, and in part by its need to be addressed through a long-term approach including public sector and salary reforms that go beyond the health sector.

The role of international partners is challenging, given that enabling country ownership, intersectoral and sustainable system approaches is a prerequisite to effectively addressing HRH – even more than it is in other areas to strengthen health systems. Moreover, many areas of HRH are perceived as government terrain, where the countries have to take a lead in defining priorities and targets that may be technically supported by international partners.

An important prerequisite for a broader involvement of bilateral and multilateral donor support to HRH appears to be donor coordination and sustained funding. As a tool to achieve the latter, funding mechanisms such as SWAps as well as the Global Fund are gaining attention. It may need flexibility and alignment among donors to facilitate such approaches.

In Malawi, for example, the costing framework of the SWAp did not initially include HRH and needed to be adapted to account for it. The Global Fund also demonstrated some flexibility in shifting funding from HIV to HRH. One perception is that it first needs targets defined at national and district level that may be supported by technical contributions from international actors.

Despite the complexity of addressing HRH, the examples above illustrate that development partners can play different roles according to their comparative advantage. The potential of German development cooperation, for example, appears to be linked to its different institutions and their ability to support training and teaching facilities, placing external staff at local rates, as well as facilitating reintegration. Moreover, a frequently perceived advantage of this bilateral agency is its representation at national and district level.

A message drawn from this analysis is that international partners do face challenges to address HRH, but overcoming those is very much in line with promoting sustainable and sector-wide approaches. Some countries and partners have started to do so, for example, by capitalizing on funding mechanisms such as SWAps and the Global Fund. But even those countries still have a multitude of parallel programmes and partners that continue traditional single approaches to training. Working towards promoting more integrated efforts appears a necessity in order to close the gap between what is stated at international policy level and what is implemented at country level.

## Competing interests

The authors declare that they have no competing interests.

## Authors' contributions

RM has analysed the data, conceptualized and written the manuscript, and was involved in the original acquisition of data. KW has critically revised the manuscript for intellectual content and was critically involved in the original study concept and acquisition of data. HP was substantially involved in the original acquisition of data and contributed to drafting the manuscript.
